# PRMT5/Sohlh2/Sirt1 Signaling Pathway in Vascular Endothelial Cells Modulates Lung Metastasis of Triple‐Negative Breast Cancer

**DOI:** 10.1002/advs.74884

**Published:** 2026-03-23

**Authors:** Ruihong Zhang, Fengqin Wang, Lanlan Liu, Qi Zhang, Xiaoning Huo, Yanhui Nan, Yanli Dong, Yunling Xiao, Jing Hao

**Affiliations:** ^1^ Key Laboratory of The Ministry of Education for Experimental Teratology, Department of Histology and Embryology, School of Basic Medical Sciences, Cheeloo College of Medicine Shandong University Jinan China; ^2^ Advanced Medical Research Institute Shandong University Jinan China; ^3^ Department of Geriatric Medicine Qilu Hospital of Shandong University Jinan China

**Keywords:** Lung metastasis, PRMT5, Sohlh2, TNBC, Vascular endothelial cell

## Abstract

Lung metastasis accounts for the majority of deaths in patients with triple‐negative breast cancer (TNBC). The interaction between tumor cells and vascular endothelial cells (VECs) plays a crucial role during cancer metastasis. Sohlh2 is known as a tumor suppressor gene in the breast cancer progression. However, the role of Sohlh2 in the crosstalk of VECs and tumor cells remain unclear. The results of tissue microarray show that low Sohlh2 expression in VECs is associated with poor prognosis, and metastasis in clinical TNBC patients. Functional studies reveal that overexpression Sohlh2 in VECs could repress the adhesion and trans‐endothelial migration of TNBC cells, attenuate EC senescence, increased permeability, and inflammation induced by TNBC cells. Endothelial cell‐specific knock in Sohlh2 suppresses TNBC lung metastasis. Moreover, mechanism studies show that Sohlh2 represses NF‐κB signaling pathway activation by promoting Sirt1 transcription in VECs. PRMT5‐mediated Sohlh2 arginine methylation promotes the CUL4B‐mediated ubiquitylation and degradation of Sohlh2, leading to the inhibition of Sohlh2 effects in VECs. Taken together, these findings demonstrate that PRMT5/Sohlh2/Sirt1 signaling pathway in VECs plays a critical role in regulating the lung metastasis in TNBC, indicating PRMT5/Sohlh2/Sirt1 signaling as potential targets in the treatment of TNBC metastasis.

## Graphical abstract

The cover image shows that vascular endothelial cells play an important role in lung metastasis of TNBC. Endothelial Sohlh2 acts as a gatekeeper against TNBC lung metastasis by limiting endothelial activation and tumor cell passage. PRMT5 reduces Sohlh2 stability, weakening this protective barrier. Targeting the PRMT5–Sohlh2–Sirt1 axis may offer a strategy to prevent metastatic spread.

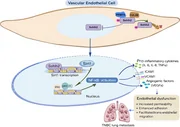

## 1 Introduction

Triple‐negative breast cancer (TNBC) refers to a specific subtype of breast cancer that lacks of the expression of estrogen receptor (ER), progesterone receptor (PR), and human epidermal growth factor receptor 2 (HER2), accounting for 15%–20% of total breast cancer cases [1, 2]. Unlike the other subtypes of breast cancer, TNBC is highly invasive, histologically highgrade, and prone to early recurrence and metastasis [3, 4]. Due to the lack of precise treatment targets, chemotherapy and adjuvant therapy are less effective, resulting in poor prognosis for TNBC. Compared with other types of breast cancer, TNBC is more prone to lung metastasis [5, 6]. Therefore, elucidating the molecular mechanisms of TNBC lung metastasis and blocking TNBC lung metastasis are urgent clinical issues to be addressed.

Metastasis of breast cancer is a complex process involving the transformation of tumor cells from the primary site to mesenchymal epithelium [7]. Under the synergistic action of cytokines secreted by tumor cells and infiltrating immune cells, tumor cells traverse the lymphatic or vascular walls, either via lymphatic circulation or direct entry into the bloodstream. Tumor cells that survive in the bloodstream will penetrate the blood vessel wall again, reach the distant metastatic site, and continuously proliferate there to form metastatic tumors [8, 9]. Endothelial cells (ECs) play a pivotal role in the tumor microenvironment and metastatic foci. The interaction between ECs and tumor cells is a decisive factor in tumor metastasis, including tumor cell adhesion to ECs, destruction of endothelial barrier function, and trans‐endothelial migration into deeper tissues [8]. However, the molecular regulatory mechanisms underlying these interactions remain incompletely understood.

Recent studies have confirmed that tumor cells secrete and release inflammatory cytokines such as IL‐1β, IL‐6; chemokines such as CCL1, CCL2; and adhesion molecules such as ICAM‐1, through different signaling pathways, activating the inflammatory response of VECs, increasing the senescence and permeability of vascular endothelium, and thus promoting tumor metastasis [8, 10, 11]. Previous study indicates that crocin inhibits angiogenesis and colorectal cancer cell metastasis by targeting NF‐κB and blocking the TNFα/NF‐κB/VEGF signaling pathway [12]. Overexpression of the transcription factor c‐Myb in breast cancer cells reduces the secretion of CCL2 by circulating tumor cells and inhibits the activation of the CCR2 signaling pathway in VECs, thereby reducing lung metastasis of breast cancer cells [13].

Sirt1 is a nicotinamide adenine dinucleotide (NAD+) dependent deacetylase that regulates gene expression through histone or non‐histone deacetylation modification, such as p53, HIF‐α, NF‐κB, FOXO3a and PPARγ, regulating cellular behaviors, such as cycle, energy consumption, oxidative stress, apoptosis [14, 15, 16]. Increasing studies indicate the specific overexpression of Sirt1 in VECs inhibits oxidative stress and high glucose‐induced EC senescence and age‐dependent vascular aging [17, 18]. Sirt1 represses the transcriptional activity of the NF‐κB complex by deacetylating the lysine 310 site of the NF‐kB subunit RelA/p65 [19]. Activation of NF‐κB signaling pathway up‐regulates VEGFa expression, increases vascular endothelial permeability, promotes inflammatory cell infiltration and tumor cell metastasis [20, 21]. Whether the vascular endothelial Sirt1/NF‐κB signaling pathway is involved in the regulation of lung metastasis in breast cancer has not been reported. Therefore, inhibiting the inflammatory response of VECs induced by tumor cells and strengthening the barrier function of vascular endothelium may be a new approach to treat tumor metastasis.

Sohlh2 is a member of the basic helix‐loop‐helix (bHLH) transcription factor family, which includes genes such as myoD, c‐Myc, and Twist. These transcription factors could bind to E‐box (CANNTG) sites of downstream target genes, regulating their expression and participating in cell proliferation, differentiation, migration, and other cellular behaviors, making them crucial genes in embryonic development and tumor initiation [22]. Our previous research has shown that Sohlh2 acts a tumor suppressor via suppressing angiogenesis, and inhibiting cell proliferation and EMT [23, 24]. Sohlh2 also functions as a oncogene to promote TNBC progression by enhancing M2 macrophage polarization [25]. It seems that Sohlh2 has dual roles in TNBC progression in a cellular context dependent manner. Recently, we found that Sohlh2 was expressed in VECs, and showed a significantly low expression in the blood vessels of TNBC metastatic tissues, suggesting that Sohlh2 may be involved in the regulation of TNBC lung metastasis.

In the present study, we demonstrated the roles of Sohlh2 in the crosstalk between VECs and TNBC cells and TNBC lung metastasis. Our work revealed that overexpression of Sohlh2 in VECs inhibited TNBC lung metastasis. Specifically, Sohlh2 restrained NF‐κB signaling pathway activation by promoting Sirt1 transcription. Moreover, we also showed that PRMT5‐mediated Sohlh2 arginine methylation promoted the CUL4B‐mediated ubiquitylation and degradation of Sohlh2. PRMT5 weakened the regulation of Sohlh2 on the interaction between VECs and TNBC cells. Our study provides a new mechanism underlying the interaction between VECs and TNBC cells mediated by Sohlh2, and shows Sohlh2 may represent a novel therapeutic target for inhibiting TNBC lung metastasis.

## 2 Results

### 2.1 Sohlh2 Expression in VECs Is Negatively Correlated with TNBC Distant Metastasis

**FIGURE 1:**
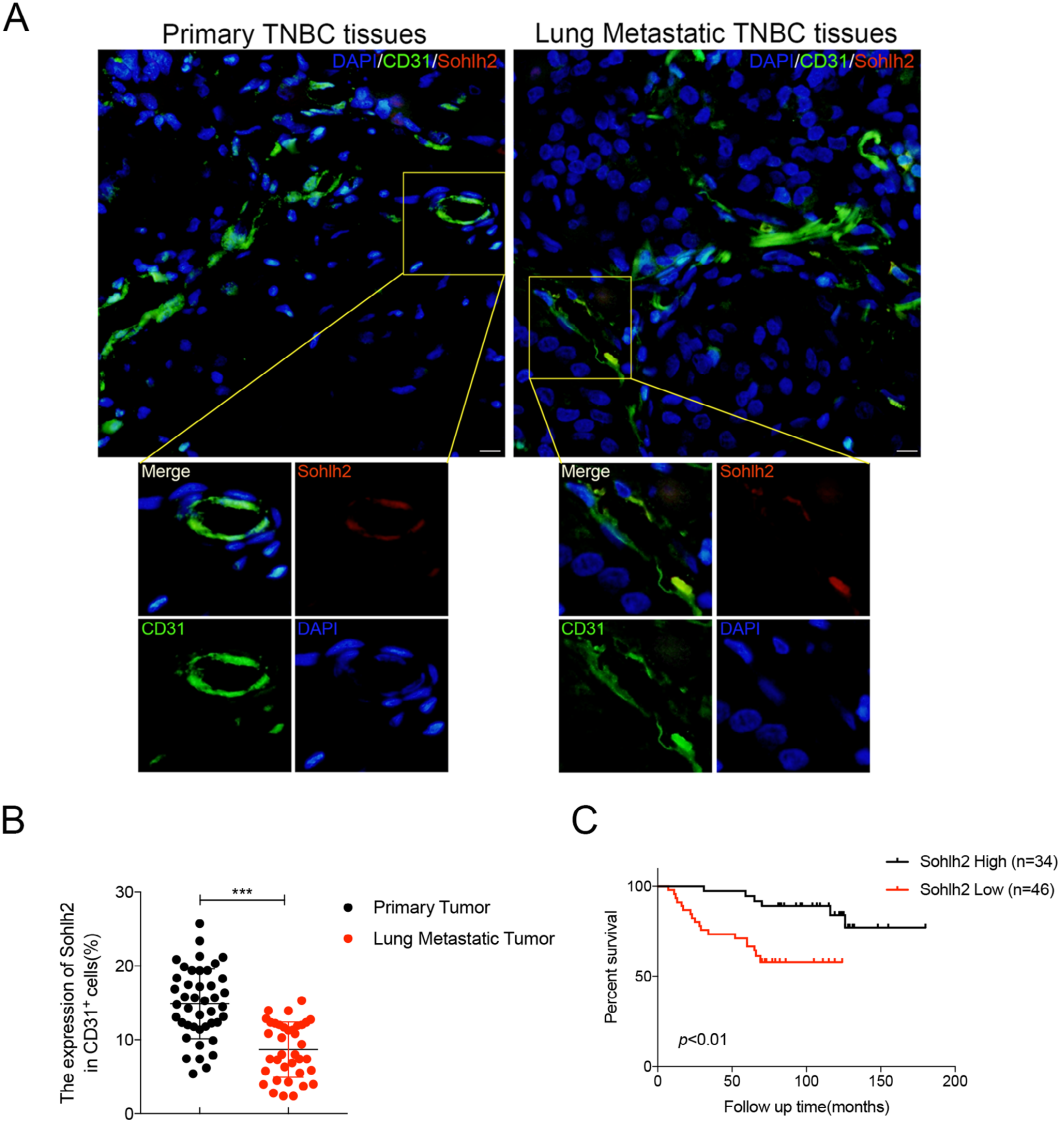
Sohlh2 expression in VECs is negatively correlated with TNBC distant metastasis. (A) Immunofluorescence assay showed the expression of Sohlh2 and CD31 in primary TNBC tissues and lung metastatic TNBC tissues (Scale bar: 20 µm). (B) The statistical result of Sohlh2 expression in CD31 positive cells (*n* = 80). (C) Kaplan–Meier survival curves depicting overall survival stratified by Sohlh2 protein expression levels of VECs in TNBC tissues. *P*‐values were calculated using the log‐rank test (*n* = 80). Data are presented as mean ± SD. An unpaired 2‐tailed *t*‐test was used in (B). ***p < 0.001.

VECs play a pivotal role in facilitating tumor distant metastasis. CD31, a member of the immunoglobulin superfamily, is a 130 kDa transmembrane glycoprotein also designated as PECAM‐1 (platelet endothelial cell adhesion molecule 1), and as a highly specific and sensitive marker for VECs, often used for visualize and identify the ECs of blood vessels [26, 27]. To investigate whether vascular endothelial Sohlh2 impacts the regulation of distant metastasis of TNBC, the relationship between vascular endothelial Sohlh2 expression and survival prognosis of TNBC patients was analyzed by CD31 and Sohlh2 immunofluorescent staining using TNBC tissue microarray. As shown in Figure 1A–C, Sohlh2 expression was significantly lower in the VECs of lung metastatic TNBC tissues than that in the primary tumor tissues, and Sohlh2 expression in VECs was positively correlated with the prognosis of TNBC patients, suggesting that endothelial Sohlh2 may serve as a potential prognostic marker in TNBC. The analysis of correlation between vascular endothelial Sohlh2 and clinical characteristics of TNBC patients showed that Sohlh2 expression was negatively correlated with tumor size, histological grade and presence of distant metastasis, and the correlation coefficient was ‐0.453, ‐0.399, and ‐0.572. However, there was no significant statistical difference with patient age (Table S1). The above results suggest that vascular endothelial Sohlh2 is negatively correlated with distant metastasis of TNBC.

### 2.2 Overexpression Sohlh2 in VECs Attenuates TNBC Cell Adhesion, Trans‐Endothelial Migration, and VEC Dysfunction Induced by TNBC Cells

**FIGURE 2:**
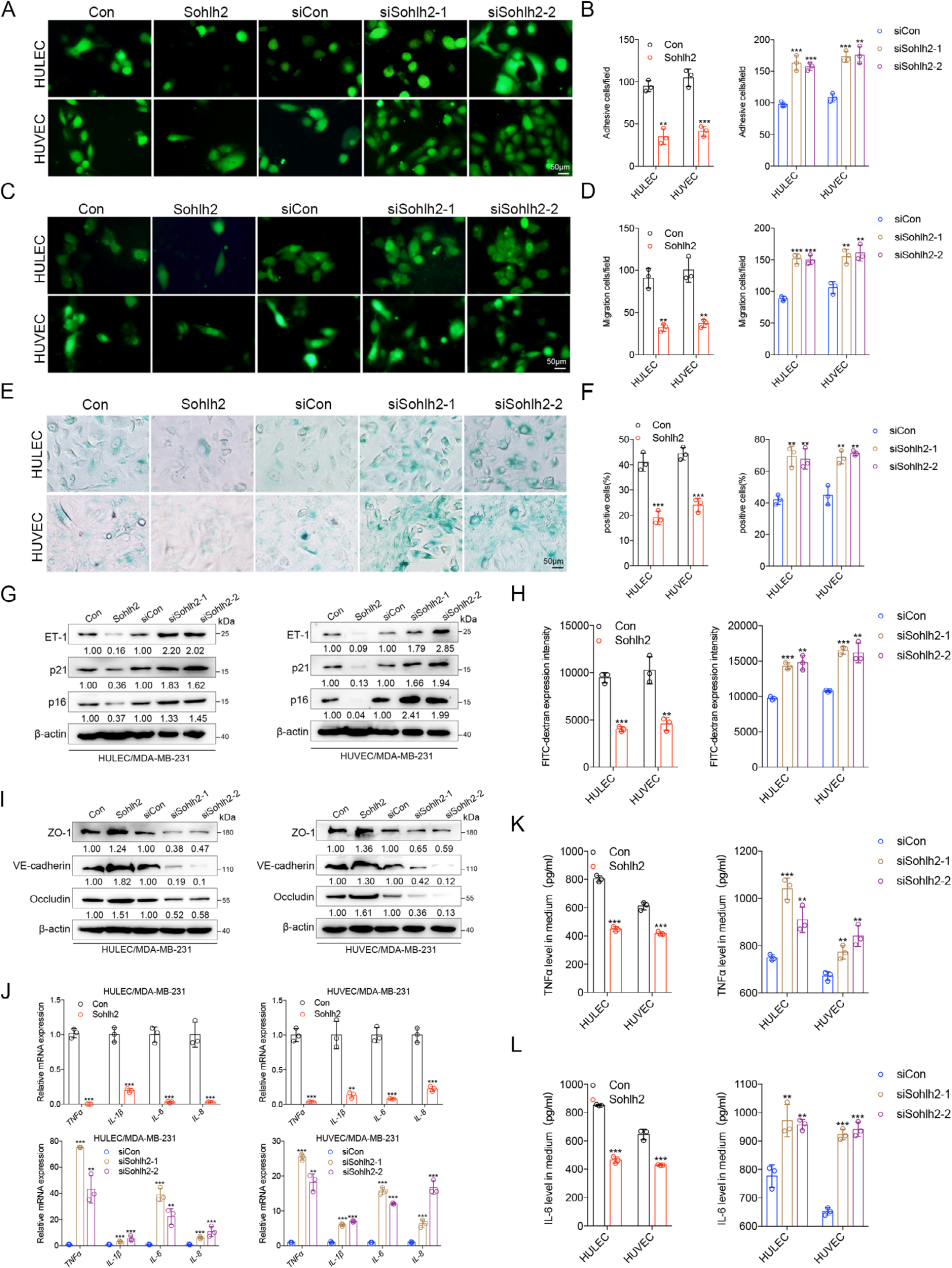
Overexpression Sohlh2 in VECs attenuates TNBC cell adhesion, trans‐endothelial migration and VEC dysfunction induced by TNBC cells. (A,B) The ability of tumor cells to adhere to VECs was examined by tumor cell adhesion assay (Scale bar: 50 µm; *n* = 3). (C,D) Trans‐endothelial migration assay was performed to detect the trans‐endothelial migration of tumor cells (Scale bar: 50 µm; *n* = 3). (E,F) SA‐β‐gal staining assay was used to examine cellular senescence (Scale bar: 50 µm; *n* = 3). (G) Senescence‐associated markers in VECs with conditioned medium (CM) from MDA‐MB‐231 cells were detected by western blot analysis. Relative protein expression levels normalized to those of β‐actin are shown below each blot. (H) Permeability experiment was carried out in VECs with CM from MDA‐MB‐231 cells (*n* = 3). (I) The tight junction proteins markers were performed by Western blot analysis. Relative protein expression levels normalized to those of β‐actin are shown below each blot. (J–L) QPCR and Elisa analysis were carried out to determine inflammatory factors TNFα, IL‐1β, IL‐6, and IL‐8 mRNA levels in VECs with CM from MDA‐MB‐231 cells (*n* = 3). Data are presented as the mean ± SD. Con and Sohlh2 were compared using the unpaired 2‐tailed *t*‐test. For siRNA experiments, differences among siCon, siSohlh2‐1, and siSohlh2‐2 were analyzed by one‐way ANOVA followed by Dunnett's post hoc multi‐comparison test (each siSohlh2 vs siCon). Experiments were repeated at least three times. **p < 0.01, ***p < 0.001.

To explore the impact of Sohlh2 in the regulation of the interaction between VECs and TNBC cells in vitro. We co‐cultured VECs with MDA‐MB‐231 cells using Transwell chamber. Sohlh2 overexpression or knockdown stable VEC lines were successfully constructed via qPCR and Western blot assay (Figure S1A–D). Tumor cell adhesion and trans‐endothelial migration assays were used to examine the effect of Sohlh2 on TNBC cell adhesion to VECs and trans‐endothelial migration. As shown in Figure 2A–D, compared to the Control (Con) group, the adhesion and trans‐endothelial migration abilities of TNBC cells were significantly repressed after overexpression Sohlh2 in VECs. While, knockdown of Sohlh2 promoted the adhesion and trans‐endothelial migration of TNBC cells compared to the siCon group (Figure 2A–D). Tumor cells secrete inflammatory cytokines, which could activate the inflammatory response of VECs and increase the senescence and permeability of VECs. Subsequently, we detected the effects of Sohlh2 on EC senescence, permeability and inflammatory response induced by TNBC cells. Cellular senescence was assessed by measuring the amounts of SA‐β‐gal positive cells and senescence markers, including p16, p21 and ET‐1. As shown in Figure 2E–G, overexpression Sohlh2 displayed significantly lower SA‐β‐gal staining compared with the Con group and decreased the protein levels of cellular senescence markers, p16, p21, and ET‐1 in VECs induced by TNBC cells. Meanwhile, knockdown Sohlh2 increased the SA‐β‐gal activity and promoted the expression of senescence protein markers (Figure 2E–G). Endothelial permeability assay results showed that Sohlh2 overexpression decreased the permeability, while Sohlh2 knockdown elevated the permeability of VECs (Figure 2H). Similarly, overexpression of Sohlh2 up‐regulated the levels of tight junction proteins VE‐cadherin, Occludin, and ZO‐1, knockdown of Sohlh2 had the opposite results (Figure 2I). QPCR and Elisa results revealed that Sohlh2 overexpression decreased the expression of VECs inflammatory factors TNFα, IL‐1β, IL‐6, and IL‐8, and knockdown Sohlh2 enhanced the levels of the above inflammatory factors (Figure 2J–L). Taken together, our data suggest that Sohlh2 overexpression suppresses EC senescence, increased permeability, and inflammation induced by TNBC cells.

### 2.3 Overexpression of Sohlh2 in VECs Inhibits TNBC Metastasis

To investigate the function of Sohlh2 in vascular endothelium, we established a mouse model enabling conditional overexpression of Sohlh2 specifically in VECs. This was achieved by crossing genetically modified mice carrying a loxP‐flanked STOP cassette followed by *Sohlh2* cDNA with *Tie2^CreERT^* mice (Figure S2A). Tamoxifen administration induced Cre‐mediated excision of the STOP cassette, leading to endothelial‐specific expression of Sohlh2 in mice of the genotype *Sohlh2^fl/fl^Tie2^Cre^* ^ERT+^ (hereafter designated Sohlh2 CKI mice). The specific expression of Sohlh2 in VECs was confirmed by genotyping (Figure S2B) and Immunofluorescence staining (Figure S2C,D), Western blot analysis (Figure S2E) and qPCR analysis (Figure S2F). Control mice consisted of *Sohlh2^fl/fl^Tie2* ^CreERT−^ littermates. Collectively, these results demonstrate the successful generation of a vascular endothelium‐specific Sohlh2 knock‐in mouse model.

**FIGURE 3:**
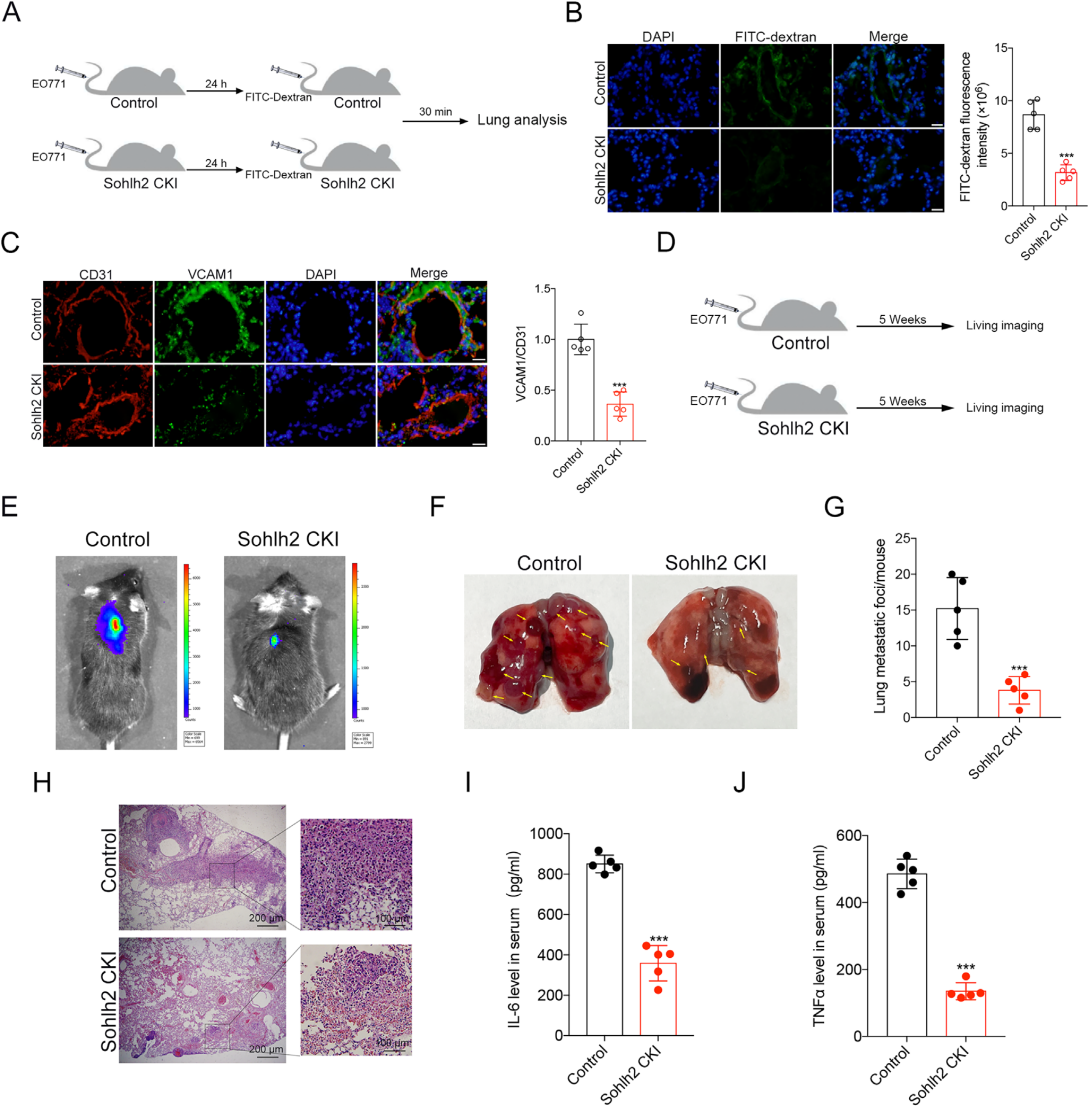
Overexpression of Sohlh2 by VECs inhibits TNBC metastasis. (A) Schematic diagram of permeability detection in vivo. (B) Analysis of FITC immunofluorescence intensity in lung tissue (Scale bar: 50 µm; *n* = 5). (C) Immunofluorescence staining of pulmonary vascular endothelial CD31 and VCAM1 (Scale bar: 50 µm; *n* = 5). (D) Schematic diagram of TNBC lung metastasis after EO771‐luciferase cells were injected into the tail vein. (E) Lung metastasis was shown by live imaging in mice. (F–H) Lung metastatic lobules were shown and detected by HE. Scale bar: 200 µm (left), 100 µm (right). (I, J) Elisa analysis was carried out to detect inflammatory factors TNFα and IL‐6 levels in serum (*n* = 5). Data are presented as the mean ± SD. Significance was determined by the unpaired 2‐tailed *t*‐test to detect differences between the groups. Experiments were repeated at least three times. ***p < 0.001.

To further evaluate the effect of vascular endothelial Sohlh2 on the permeability of pulmonary vascular endothelium in vivo, we injected FITC‐dextran into the tail vein 24 h after injecting EO771 cells (Figure 3A). 30 min later, the lung tissue was fixed for subsequent section staining. The fluorescence intensity of FITC was analyzed and immunofluorescence staining of VCAM1 in pulmonary vessels was performed. The fluorescence intensities of FITC and VCAM1 were weakened in the Sohlh2 CKI group (Figure 3B,C). To dissect further the effects of vascular endothelial Sohlh2 on TNBC lung metastasis in vivo, a TNBC lung metastasis model was established by injecting EO771‐luciferase cells into Sohlh2 CKI mice (Figure 3D). Live imaging and HE staining showed that vascular endothelial Sohlh2 overexpression inhibited TNBC lung metastasis in Sohlh2 CKI mice compared with the Control mice (Figure 3E–H). Elisa results showed that Sohlh2 decreased the serum levels of inflammatory cytokines TNFα and IL‐6 (Figure 3I,J). The above results strongly demonstrate that Sohlh2 overexpression in VECs suppresses TNBC lung metastasis.

### 2.4 Sohlh2 Enhances the Transcription of Sirt1 in VECs and Restrains the Activation of NF‐κB Signaling Pathway

**FIGURE 4:**
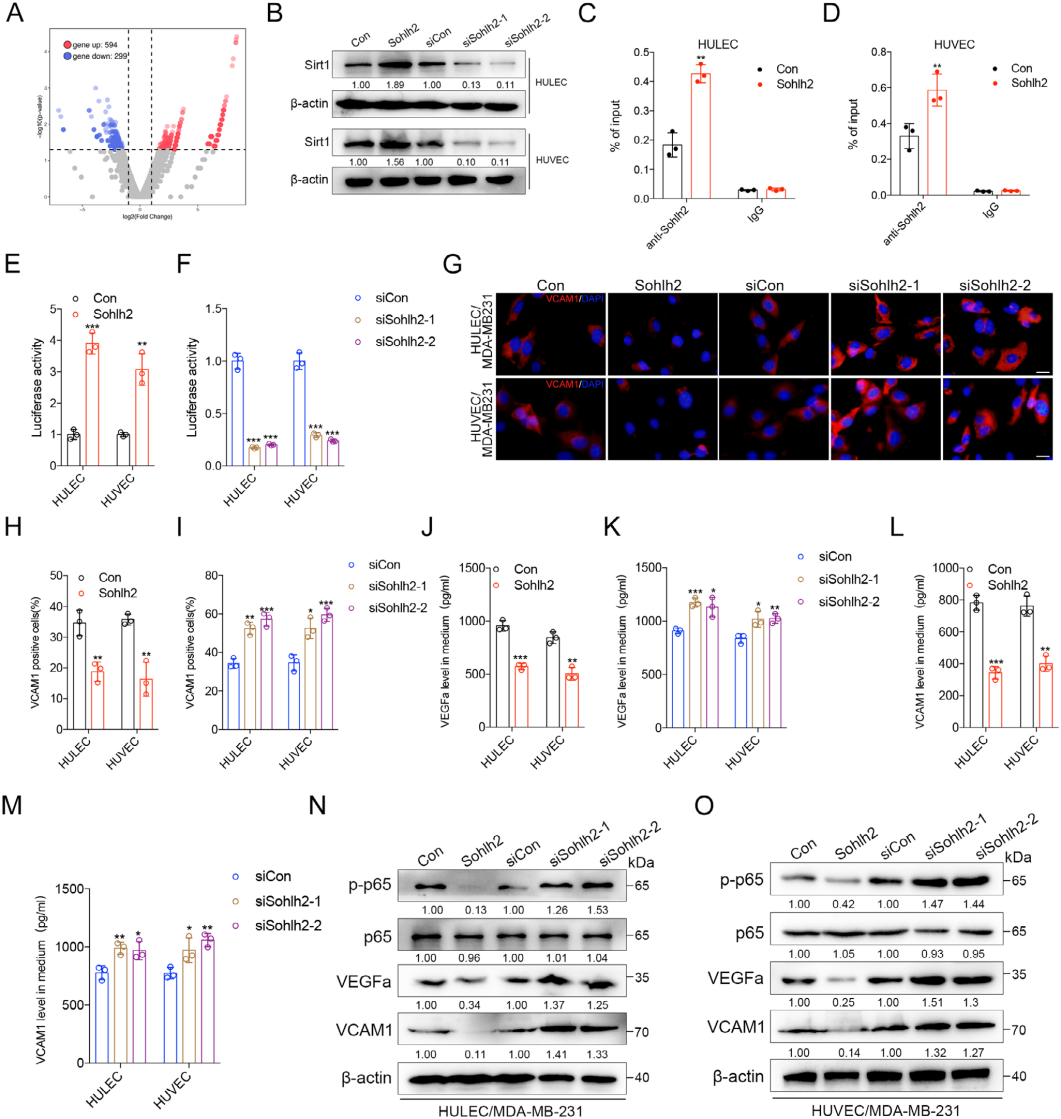
Sohlh2 enhances the transcription of Sirt1 in VECs and restrains the activation of NF‐κB signaling pathway. (A) Volcanic map showed the changes in gene expression in the Con vs. Sohlh2 HULECs. The threshold was set to |log2 (fold change)| ≥1 and *Q* value < 0.05. (B) Western blot analysis was performed to determine the Sirt1 protein level in VECs. Relative protein expression levels normalized to those of β‐actin are shown below each blot. (C,D) ChIP analysis of forced Sohlh2 expression VECs using anti–Sohlh2 antibody for Sirt1 promoter (*n* = 3). (E,F) Sirt1 promoter luciferase reporter activity in Sohlh2 overexpression or knockdown VECs (*n* = 3). (G–I) Immunofluorescence staining was used to detect the expression of VCAM1 in VECs with CM from TNBC cells (Scale bar: 50 µm; *n* = 3). (J–M) Elisa analysis was carried out to detect VEGFa and VCAM1 levels in culture medium (*n* = 3). (N,O) Western blot assay showed the expression of VEGFa, VCAM1, and p‐p65 in Sohlh2 overexpression or knockdown VECs with CM from TNBC cells. Relative protein expression levels normalized to those of β‐actin are shown below each blot. Data are presented as the mean ± SD. An unpaired 2‐tailed *t*‐test was used in (C–E,H,J,L). One‐way ANOVA followed by Dunnett's post hoc multi‐comparison test was used in (F,I,K,M). Experiments were repeated at least three times. *p < 0.05, **p < 0.01, ***p < 0.001.

To further assess the mechanism of VEC Sohlh2 regulating TNBC lung metastasis, the Con and Sohlh2 HULECs were treated with MDA‐MB‐231 cell CM, and RNA was extracted for RNA sequencing. RNA‐seq results showed that 594 genes were upregulated and 299 genes were downregulated in the Sohlh2 group, and Sohlh2 upregulated Sirt1 expression at 8.33‐fold (Figure 4A; Figure S3A). As shown in Figure S3B, Sirt1 mRNA levels were increased by Sohlh2, knockdown of Sohlh2 downregulated Sirt1 mRNA levels. Similarly, we evaluated the changes in protein levels of Sirt1 by Western blot analysis (Figure 4B). Furthermore, through immunofluorescence staining of the pulmonary vascular endothelium of Sohlh2 CKI mice, it was found that compared with the control group, the expression of Sirt1 was increased in the Sohlh2 CKI group (Figure S3C). Subsequently, ChIP and dual luciferase reporter assay were performed to further investigate the relationship between Sohlh2 and the Sirt1 promoter. As shown in Figure 4C–F, Sohlh2 could bind to the promoter region of Sirt1 and increase its transcriptional activity, while knockdown of Sohlh2 restrained its transcriptional activity. Since it had been reported that Sirt1 prevented NF‐κB signaling pathway activation by deacetylating p65 protein, then decreased the expression of multiple proinflammatory cytokines in VECs, and significantly reduced the endothelial dysfunction caused by inflammation [18, 19, 20, 21]. As expected, we enriched the NF‐κB signaling pathway in Sohlh2 RNAi VECs by PCR Array (Figure S3D,E). Immunofluorescence staining showed that Sohlh2 reduced the VCAM1 positive cells, knockdown Sohlh2 increased the VCAM1 positive cells (Figure 4G–I). Elisa and qPCR results showed that overexpression Sohlh2 decreased the levels of VEGFa and VCAM1 in VECs, knockdown Sohlh2 had the opposite results (Figure 4J–M; Figure S3F). Consistent with the aforementioned data, overexpression Sohlh2 exhibited lower protein levels of VEGFa, VCAM1 and p‐p65 (Figure 4N,O). Immunofluorescence assay also showed that overexpression Sohlh2 reduced the protein level of p‐p65 in VECs (Figure S3G). Taken together, these findings demonstrate that VEC Sohlh2 enhances the transcription of Sirt1 and suppresses the activation of the NF‐κB signaling pathway.

### 2.5 Sirt1 Mediates the Effects of Sohlh2 on the Interaction Between VECs and TNBC Cells

**FIGURE 5:**
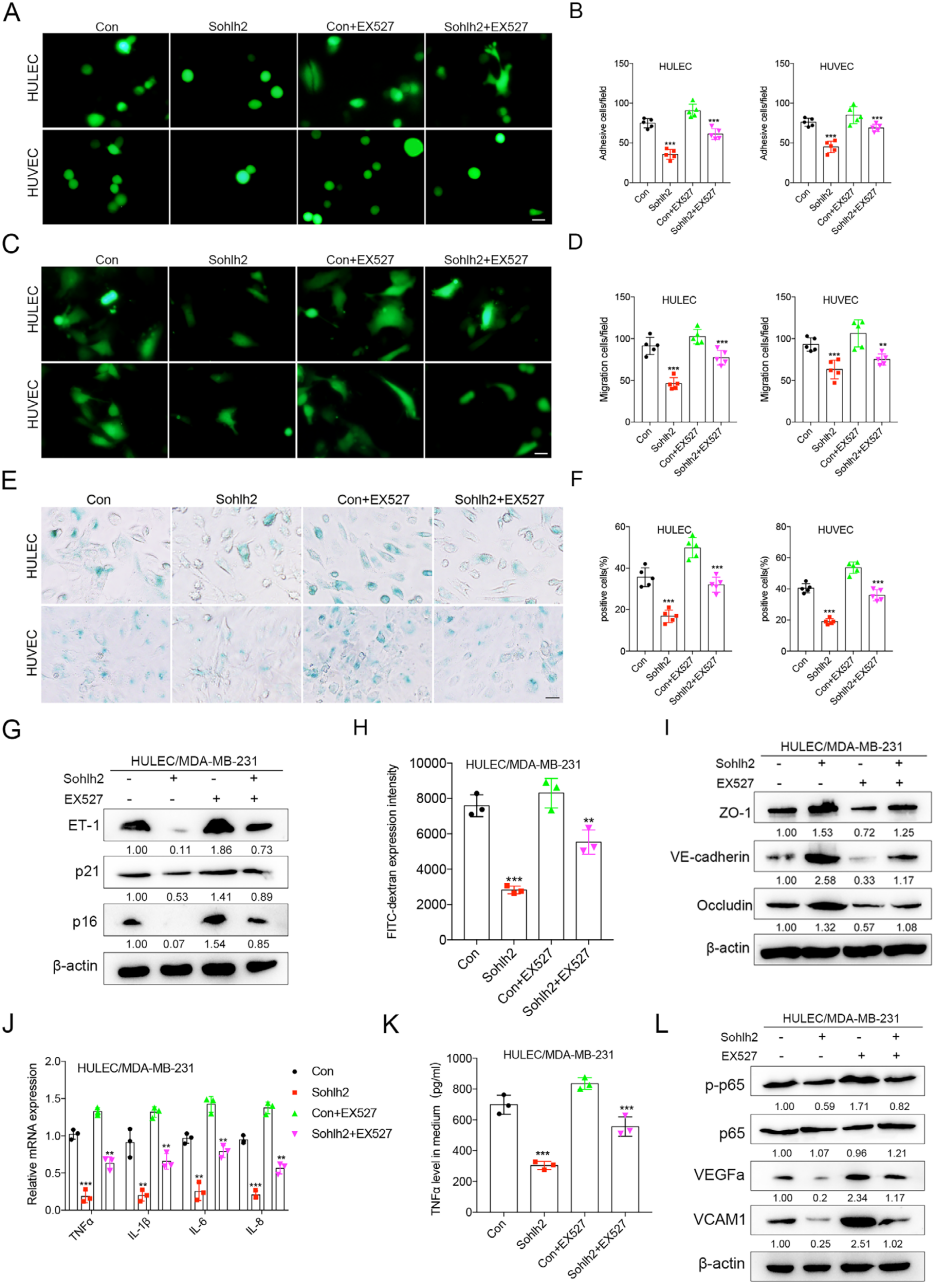
Sirt1 mediates the effects of Sohlh2 on the interaction between VECs and TNBC cells. (A,B) The adhesion ability of MDA‐MB‐231 cells was examined in Sohlh2 overexpression VECs with EX527 treatment by tumor cell adhesion assay (Scale bar: 50 µm; *n* = 3). (C,D) Trans‐endothelial migration assay showed that the TNBC cell number of trans‐endothelial migration in Sohlh2 overexpression VECs with EX527 added (Scale bar: 50 µm; *n* = 3). (E–G) SA‐β‐gal staining and Western blot analysis were carried out to detect the senescence of Sohlh2 overexpression VECs with EX527 treatment (Scale bar: 50 µm; *n* = 3). Relative protein expression levels normalized to those of β‐actin are shown below each blot. (H) Permeability experiment was carried out in Sohlh2 overexpression VECs with CM from MDA‐MB‐231 cells after EX527 added (*n* = 3). (I) The tight junction proteins markers were investigated in Sohlh2 overexpression VECs with EX527 cultured with CM from MDA‐MB‐231 cells by Western blot analysis. Relative protein expression levels normalized to those of β‐actin are shown below each blot. (J,K) QPCR and Elisa analysis were performed to determine the expression of inflammatory cytokines in Sohlh2 overexpression VECs with EX527 cultured with CM from MDA‐MB‐231 cells (*n* = 3). (L) Western blot analysis showed the protein levels of p‐p65, VEGFa, and VCAM1 in Sohlh2 overexpression VECs with CM from MDA‐MB‐231 cells after EX527 was added. Relative protein expression levels normalized to those of β‐actin are shown below each blot. Data are presented as the mean ± SD. One‐way ANOVA analysis followed by Sidak post hoc multi‐comparison test was used. Experiments were repeated at least three times. **p < 0.01, ***p < 0.001.

In order to further clarify whether Sirt1 mediates the role of Sohlh2 on the interaction between VECs and TNBC cells. EX527, a selective inhibitor of Sirt1 was added in the Sohlh2 overexpressing VECs with CM from TNBC cells. Interestingly, tumor cell adhesion assay and trans‐endothelial migration assay showed that the number of adhered and trans‐endothelial migrated TNBC cells in the EX527 plus Sohlh2 group was significantly more than the Sohlh2 group (Figure 5A–D). Surprisingly, SA‐β‐gal‐positive cells were remarkably more abundant following EX527 administration (Figure 5E,F). These results were further confirmed by Western blot analysis, which showed that EX527 significantly increased p16, p21, and ET‐1 protein levels in Sohlh2 overexpressing VECs (Figure 5G). Moreover, the permeability of VECs was increased with EX527 addition, and EX527 decreased the protein levels of tight junction proteins VE‐cadherin, Occludin, and ZO‐1 in comparison with Sohlh2 overexpressing group (Figure 5H,I). As shown in Figure 5J,K, qPCR and Elisa analysis showed that EX527 promoted the expression of inflammatory cytokines, such as, TNFα, IL‐1β, IL‐6, and IL‐8 compared with the Sohlh2 group. Furthermore, the protein levels of p‐p65 and NF‐κB signaling target genes VEGFa and VCAM1 were up‐regulated in the Sohlh2 group treated with EX527 (Figure 5L). At the same time, SRT1720, a selective activator of Sirt1 was treated with VECs knockdown Sohlh2, as expected, in the SRT1720+ siSohlh2‐1 group, SRT1720 treatment reduced the adhesion and trans‐endothelial migration of TNBC cells compared to the siSohlh2‐1 group (Figure S4A–D). Consistently, SRT1720 attenuated the senescence, permeability and inflammatory response of VECs in comparison with the siSohlh2‐1 group (Figure S4E–L). In addition, we also constructed the knockdown plasmid of Sirt1, and transfected it on the basis of overexpression of Sohlh2 in VECs, and detected the knockdown efficiency of Sirt1 by qPCR and Western blot assay (Figure S5A,B). The results of the tumor cell trans‐endothelial migration and adhesion assays showed that compared with the Sohlh2 group, knockdown of Sirt1 increased the adhesion and trans‐endothelial migration of TNBC cells (Figure S5C,D). Collectively, these results suggest that Sirt1 mediates the regulation of Sohlh2 on the interaction between VECs and TNBC cells.

### 2.6 Sirt1 Mediates the Inhibitory Effects of Sohlh2 on TNBC Lung Metastasis

**FIGURE 6:**
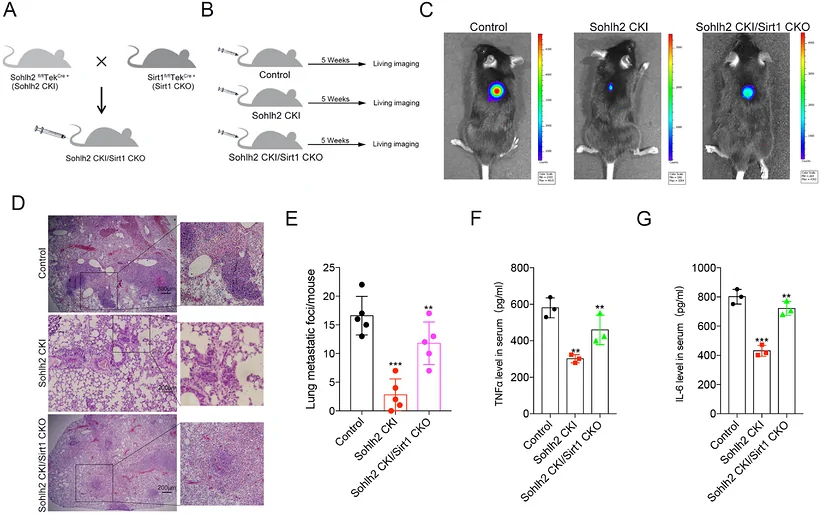
Sirt1 mediates the inhibitory effects of Sohlh2 on TNBC lung metastasis. (A) Schematic diagram depicting the generation of endothelial cell‐specific Sohlh2 knock‐in mice and knockout Sirt1. (B) Schematic diagram of TNBC lung metastasis after EO771‐luciferase cells were injected into the tail vein in Sohlh2 CKI/Sirt1 CKO mice. (C) Lung metastasis was shown by live imaging in mice. (D,E) Lung metastatic lobules were shown and detected by HE (Scale bar: 200 µm; *n* = 5). (F,G) Elisa analysis was carried out to detect inflammatory factors TNFα and IL‐6 levels in serum (*n* = 5). Data are presented as the mean ± SD. One‐way ANOVA analysis followed by Sidak post hoc multi‐comparison test was used. Experiments were repeated at least three times. **p < 0.01, ***p < 0.001.

To further verify whether Sirt1 mediates the inhibitory effect of Sohlh2 on lung metastasis of TNBC, a TNBC lung metastasis model was constructed using vascular endothelial conditional knock‐in Sohlh2 and knockout Sirt1 (Sohlh2 CKI/Sirt1 CKO) mice injected with EO771‐luciferase cells in the tail vein (Figure 6A,B). Live imaging and HE staining showed that lung metastasis were significantly more frequent in the Sohlh2 CKI/Sirt1 CKO group than in the Sohlh2 CKI group (Figure 6C‐E). Elisa and qPCR analysis showed that the serum inflammatory cytokines TNFα, IL‐1β, IL‐6, and IL‐8 were significantly high in the Sohlh2 CKI/Sirt1 CKO group compared to Sohlh2 CKI group (Figure 6F,G). Taken together, these results further suggest that Sirt1 mediates the inhibitory effect of Sohlh2 on TNBC lung metastasis.

### 2.7 Sohlh2 is Positively Correlated with Sirt1 Expression in VECs of TNBC Tissues

**FIGURE 7:**
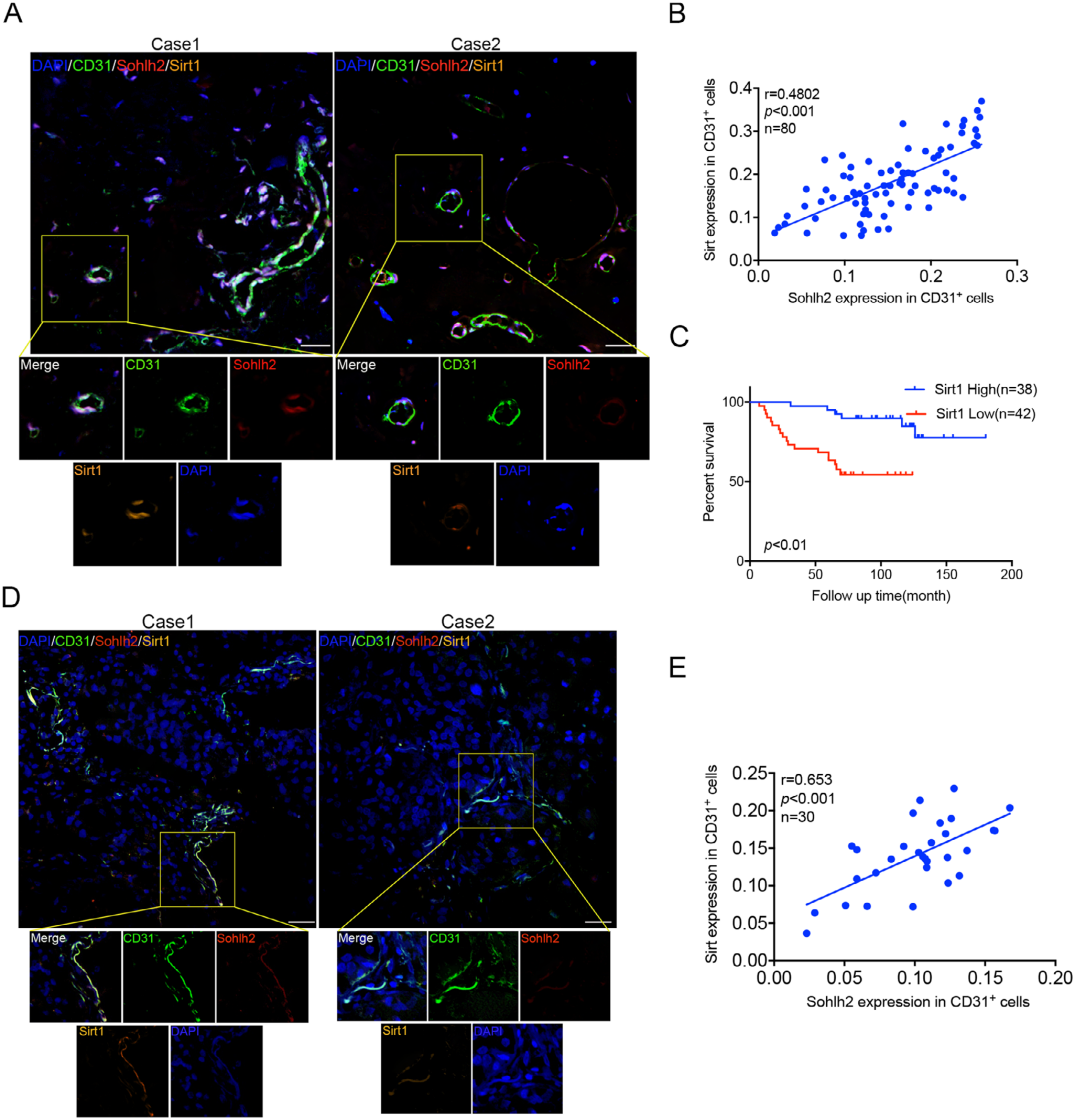
Sohlh2 expression is positively correlated with Sirt1 expression in TNBC VECs. (A) Immunofluorescence assay showed the expression of CD31, Sohlh2, and Sirt1 in TNBC tissues (Scale bar: 50 µm; *n* = 80). (B) The correlation of Sohlh2 expression and Sirt1 expression in CD31 positive cells (*n* = 80). (C) Kaplan–Meier survival curves depicting overall survival stratified by Sohlh2 protein expression levels of VECs in TNBC tissues. Statistical analysis was performed using the log‐rank test. (D) Immunofluorescence assay showed the expression of CD31, Sohlh2, and Sirt1 in lung metastatic TNBC tissues (Scale bar: 50 µm; *n* = 30). (E) The correlation of Sohlh2 expression and Sirt1 expression in CD31 positive cells (*n* = 30). All results are presented as mean ± SD. Pearson correlation test (B,E).

To investigate the correlation of Sohlh2 and Sirt1 expression in TNBC tissues, the correlation of vascular endothelial Sohlh2 with Sirt1 protein expression was analyzed by CD31, Sohlh2 and Sirt1 immunofluorescence staining using TNBC tissue microarray. Immunofluorescence staining results showed that Sohlh2 expression were positively correlated with Sirt1 expression in VECs, and Sirt1 expression in VECs was positively correlated with the prognosis of TNBC patients (Figure 7A–C). To further explore the expression of Sohlh2 and Sirt1 in lung metastasis tissues of TNBC, vascular endothelial Sohlh2 with Sirt1 protein expression was analyzed by CD31, Sohlh2, and Sirt1 immunofluorescence staining in lung metastatic TNBC tissues. As shown in Figure 7D,E, Sohlh2 expression were positively correlated with Sirt1 expression in VECs of lung metastatic TNBC tissues.

### 2.8 PRMT5 Mediates Arginine Methylation and Destabilizes Sohlh2 Protein in VECs

**FIGURE 8:**
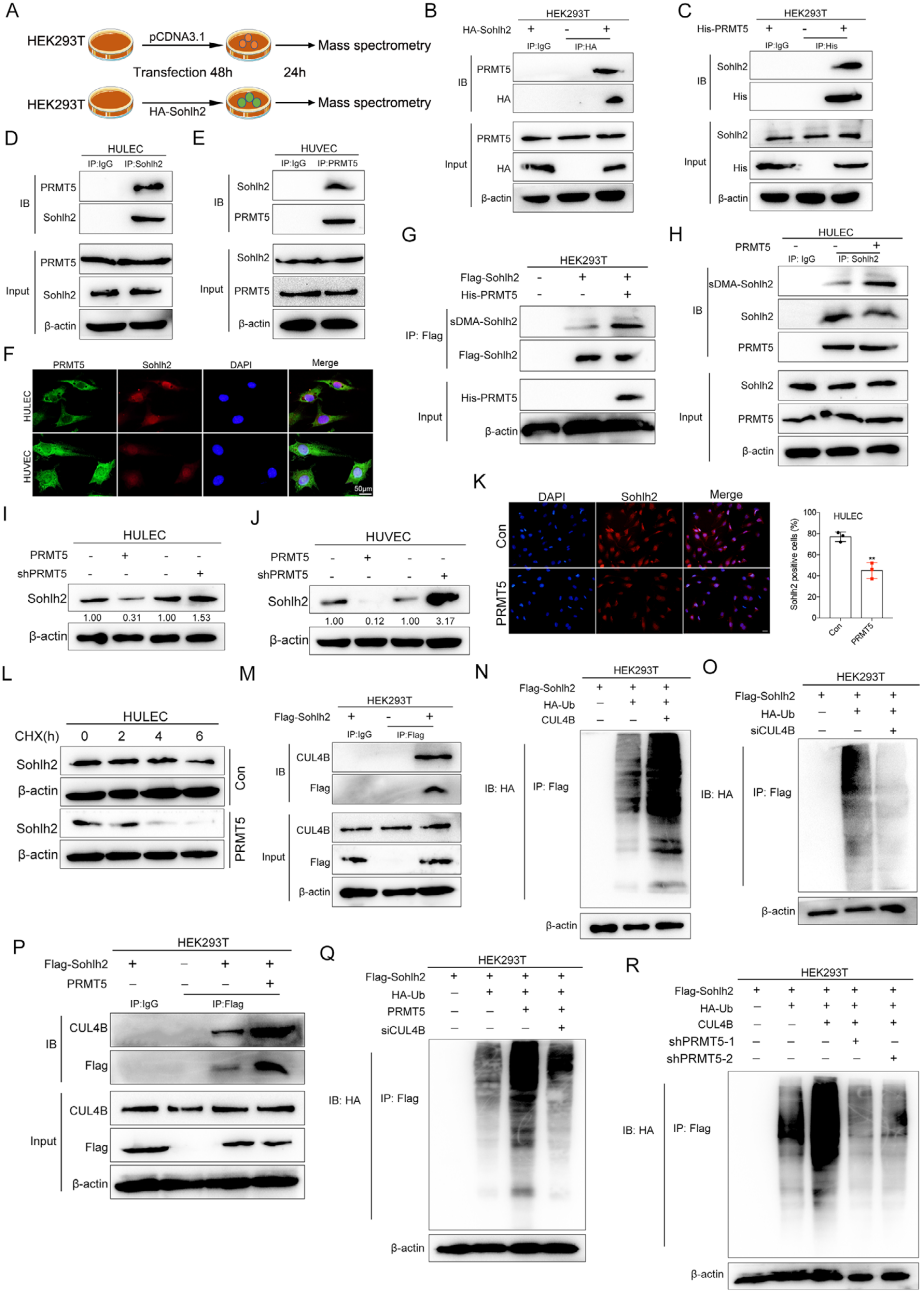
PRMT5 mediates arginine methylation modification and protein stability of endothelial Sohlh2. (A) Flow chart of mass spectrometry analysis. (B–E) Co‐IP assay was performed to detect the interaction between PRMT5 and Sohlh2. (F) The co‐localization of PRMT5 and Sohlh2 was detected by immunofluorescent analysis (Scale bar: 50 µm; *n* = 3). (G,H) Co‐IP assay showed the symmetric di‐methylation of Sohlh2. (I,J) Western blot analysis showed the expression of Sohlh2 in PRMT5 overexpression or knockdown VECs. (K) Immunofluorescence staining showed a significant decrease of Sohlh2 protein levels in PRMT5 overexpression VECs (Scale bar: 50 µm; *n* = 3). (L) Western blot analysis showed that Sohlh2 degraded via the ubiquitin‐mediated proteasome pathway in VECs were treated with CHX (50 µg/mL) for 6 h. (M) Co‐IP assay was performed to detect the interaction between CUL4B and Sohlh2. (N,O) Co‐IP assay and Western blot analysis were performed to detect the role of CUL4B on the ubiquitination of Sohlh2. (P) Co‐IP assay showed PRMT5 promoted the binding of Sohlh2 to CUL4B. (Q,R) PRMT5 promoted CUL4B‐mediated Sohlh2 ubiquitination degradation by Co‐IP assay. Data are presented as the mean ± SD. Statistical analysis were performed by GraphPad Prism 7 using the unpaired 2‐tailed *t*‐test to detect differences between the groups(K). Data are representative of three independent experiments. **p < 0.01.

A variety of studies have confirmed that post‐translational modification of proteins is involved in the regulation of protein function by altering protein activity, stability and interaction with other molecules. To explore the post‐translational modification of Sohlh2 protein, the Sohlh2‐interacting protein PRMT5 was screened by Co‐IP combined mass spectrometry (Figure 8A). Co‐IP and immunofluorescent co‐localization analysis confirmed the interaction between Sohlh2 and PRMT5 (Figure 8B–F). As shown in Figure 8G,H, PRMT5 elevated Sohlh2 symmetric di‐methylation. In order to further study the effect of PRMT5 on the stability of Sohlh2, VECs were transfected with overexpression of PRMT5 or shPRMT5 plasmid, Western blot analysis showed that overexpression of PRMT5 down‐regulated Sohlh2 protein level in VECs (Figure 8I,J). Immunofluorescence results showed that overexpression of PRMT5 decreased the expression of Sohlh2 protein (Figure 8K). Next, PRMT5 promoted the degradation of Sohlh2 protein when PRMT5 was overexpressed in VECs treated with the CHX (Figure 8L). The ubiquitin‐proteasome system plays an important role in regulating protein stability. In order to investigate the mechanism of promoting the instability of Sohlh2 protein by overexpression of PRMT5 in VECs, the Sohlh2‐interacting protein E3 ligase CUL4B was selected by Co‐IP combined mass spectrometry. As shown in Figure 8M, Sohlh2 interacted with CUL4B. HEK293T cells were transfected with Flag‐Sohlh2, HA‐Ubiquitin, overexpressed CUL4B or shCUL4B plasmid. Western blot analysis showed that CUL4B promoted the ubiquitination and degradation of Sohlh2 (Figure 8N,O). Co‐IP analysis showed that PRMT5 promoted the binding of Sohlh2 to CUL4B (Figure 8P). To further explore the effect of PRMT5‐mediated Sohlh2 methylation on the stability of Sohlh2, subsequently, HEK293T cells were immediately transfected with Flag‐Sohlh2 plasmid, HA‐Ubiquitin, shCUL4B or overexpressed PRMT5 plasmid. Co‐IP and Western blot analysis showed that PRMT5 enhanced CUL4B‐mediated Sohlh2 ubiquitination and degradation, while knockdown PRMT5 inhibited CUL4B‐mediated Sohlh2 ubiquitination and degradation (Figure 8Q,R). These results suggest that PRMT5‐mediated arginine methylation of Sohlh2 is involved in the regulation of Sohlh2 protein stability mediated by CUL4B.

### 2.9 PRMT5 Attenuates Sohlh2‐Mediated the Interaction Between VECs and TNBC Cells

**FIGURE 9:**
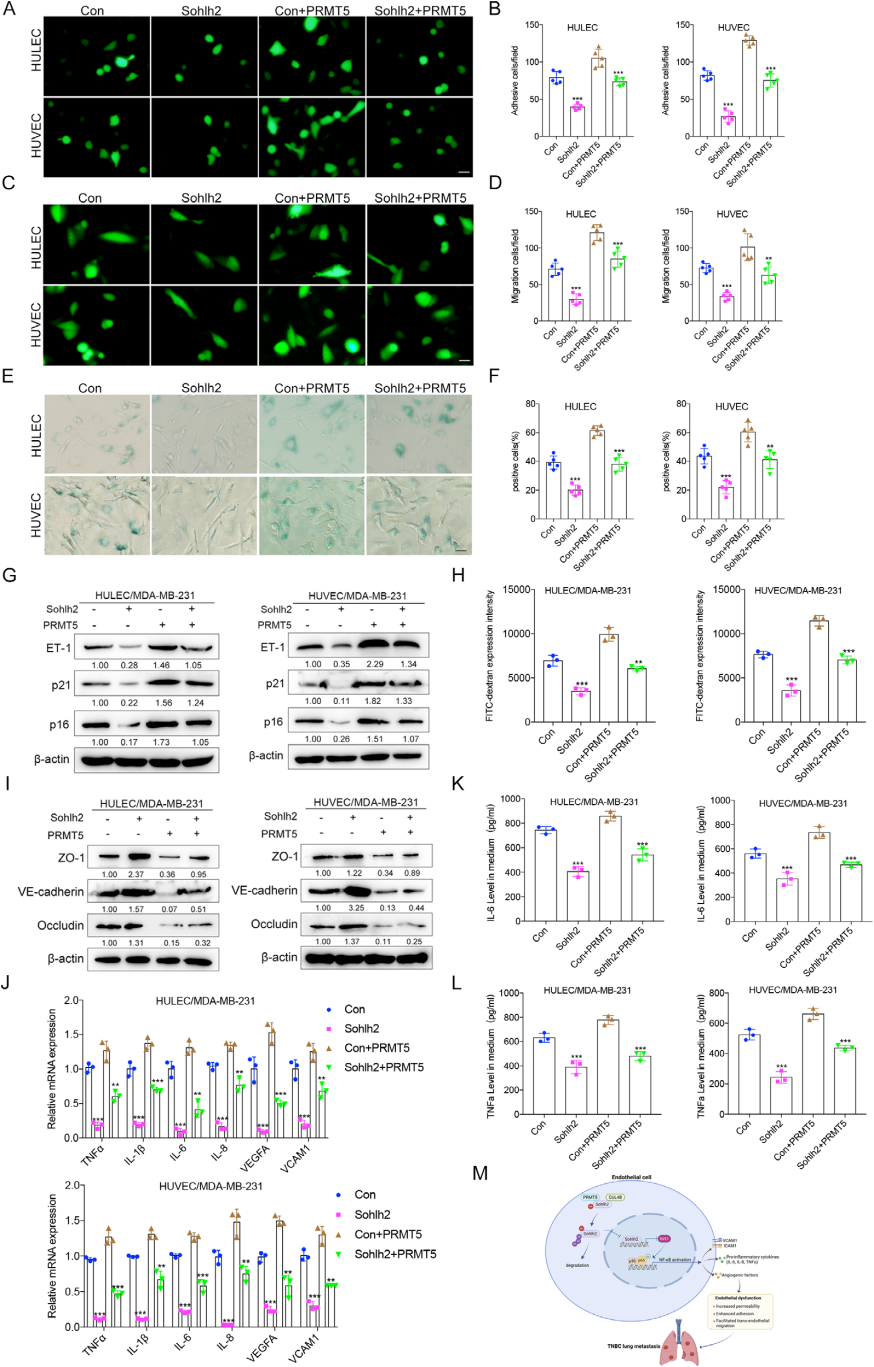
PRMT5 attenuates Sohlh2‐mediated the interaction between VECs and TNBC cells. (A,B) The adhesion ability of MDA‐MB‐231 cells was examined in Sohlh2 overexpression VECs transfected with PRMT5 plasmid by tumor cell adhesion assay (Scale bar: 50 µm; *n* = 3). (C,D) Trans‐endothelial migration assay showed that the number of TNBC cells trans‐endothelial migration VECs transfected with PRMT5 plasmid (Scale bar: 50 µm; *n* = 3). (E–G) SA‐β‐gal staining and Western blot analysis were carried out to detect the senescence of Sohlh2 overexpression VECs transfected with PRMT5 plasmid and cultured with CM from MDA‐MB‐231 cells (Scale bar: 50 µm; *n* = 3). Relative protein expression levels normalized to those of β‐actin are shown below each blot. (H) Permeability experiment was carried out in Sohlh2 overexpression VECs transfected with PRMT5 plasmid and cultured with CM from MDA‐MB‐231 cells (*n* = 3). (I) The tight junction proteins markers were performed in Sohlh2 overexpression VECs transfected with PRMT5 plasmid and cultured with CM from MDA‐MB‐231 cells by Western blot analysis. Relative protein expression levels normalized to those of β‐actin are shown below each blot. (J–L) QPCR and Elisa analysis were performed to determine the expression of inflammatory cytokines in Sohlh2 overexpression VECs transfected with PRMT5 plasmid and cultured with CM from MDA‐MB‐231 cells (*n* = 3). (M) A schematic model showed the role of the PRMT5/Sohlh2/Sirt1 signaling pathway on the interaction between VECs and TNBC cells. Data are presented as the mean ± SD. One‐way ANOVA analysis followed by Sidak post hoc multi‐comparison test was used. Experiments were repeated at least three times. **p < 0.01, ***p < 0.001.

To further study the regulation of the interaction between VECs and TNBC cells by PRMT5‐mediated Sohlh2 arginine methylation modification, PRMT5 overexpression plasmid was transfected into overexpressed Sohlh2 VECs and co‐cultured with TNBC cells. The overexpressing efficiency of PRMT5 effects was evaluated by qPCR and western blot assays (Figure S6A,B). Tumor cell adhesion and trans‐endothelial migration analysis showed that PRMT5 weakened the inhibition of Sohlh2 on TNBC cell adhesion to VECs and trans‐endothelial migration (Figure 9A–D). As shown in Figure 9E–G, PRMT5 increased SA‐β‐gal‐positive cells and upregulated the aging protein levels of p16, p21, and ET‐1 compared to Sohlh2 group. Subsequently, the permeability of VECs was performed, and the results showed that the VEC permeability in the PRMT5 plus Sohlh2 group was significantly increased (Figure 9H). Consistently, PRMT5 repressed the tight junction protein levels of VE‐cadherin, Occludin and ZO‐1 in comparison with the Sohlh2 group (Figure 9I). Meanwhile, qPCR and Elisa analysis showed that the expression of inflammatory factors TNFα, IL‐1β, IL‐6 and IL‐8 in the PRMT5 plus Sohlh2 group was higher than that in the Sohlh2 group (Figure 9J–L). A schematic model showed the role of the PRMT5/Sohlh2/Sirt1 signaling pathway on the interaction between VECs and TNBC cells (Figure 9M). Taken together, these results suggest that PRMT5 weakens the regulation of Sohlh2 on the interaction between VECs and TNBC cells.

## 3 Discussion

Emerging evidences have confirmed that VECs play a key role in the distant metastasis of TNBC [8, 9]. In our study, we revealed that the expression of vascular endothelial Sohlh2 in TNBC tissues was negatively correlated with TNBC metastasis, suggesting that vascular endothelial Sohlh2 might be involved in the regulation of TNBC metastasis.

The interaction between tumor cells and VECs plays a crucial role in the distant metastasis of tumors. During the process of tumor metastasis, factors affecting endothelial cell permeability, adhesion molecule secretion, inflammatory response, and aging can all participate in the regulation of tumor metastasis [28, 29, 30]. Tumor cells could induce inflammatory responses in pulmonary VECs, activate the NF‐κB signaling pathway, increase the permeability of ECs, cause senescence, and change the expression of adhesion molecules, promote the adhesion between tumor cells and ECs and trans‐endothelial migration, which is conducive to the pulmonary implantation of TNBC cells and the formation of metastases. The barrier function of VECs depends on the expression of VE‐cadherin in VECs [31]. VE‐cadherin is an endothelial cell‐specific adhesion linker protein that regulates the stability of the EC barrier by interacting with catenin and actin cytoskeleton [32]. Trans‐endothelial migration of tumor cells requires disruption of endothelial adhesion connections formed by VE‐cadherin. In this study, we confirmed that overexpression Sohlh2 in VECs inhibited lung metastasis of TNBC by increasing the levels of VE‐cadherin, decreasing the expression of VEGFa, and reducing the increase of VECs permeability induced by TNBC cells. In addition, tumor cells adhere to VECs, prompting VECs to secrete adhesion molecules, such asICAM1 and VCAM1, to increase the adhesion between tumor cells and VECs, resulting in tumor invasion and metastasis [33]. VCAM1 is a adhesion molecule that is closely related to EC inflammation and dysfunction. VCAM1 in VECs is involved in the regulation of distant tumor metastasis by promoting the adhesion of tumor cells to VECs [34]. In our work, we confirmed that the overexpression Sohlh2 in VECs down‐regulated the expression of VCAM1, inhibited the adhesion of TNBC cells to VECs, and weakened the inflammatory response and senescence of VECs induced by TNBC cells. Moreover, TNBC lung metastasis in Sohlh2 CKI mice were significantly reduced compared with the control group. These results suggest that vascular endothelial Sohlh2 may be an important target for TNBC lung metastasis therapy.

Sirt1, as a deacetylase, plays a crucial role in the homeostasis of VECs. The role of Sirt1 in lung metastasis of TNBC has not been reported. In our study, RNA‐seq results showed Sirt1 was significantly up‐regulated in overexpressed Sohlh2 VECs. Previous research reported that Sirt1 inhibited the transcriptional activity of the NF‐κB complex by deacetylating the lysine 310 site of the NF‐kB subunit RelA/p65 [19]. Downstream target genes of NF‐κB include IL‐8, VEGFa, VCAM1, which are involved in the regulation of EC inflammation [35]. Activation of NF‐κB signaling pathway in VECs plays an important role in tumor metastasis. Subsequently, we confirmed that Sohlh2 enhanced the transcription of Sirt1 in VECs, suppressed the activation of NF‐κB signaling pathway. In addition, Sohlh2 CKI/Sirt1 CKO mice showed more severe lung metastasis than Sohlh2 CKI mice. ChIP and dual luciferase reports validated that Sohlh2 directly bound to the promoter region of Sirt1 and promoted its transcriptional activity. These results suggest that Sirt1 is the target gene of transcription factor Sohlh2. To further ascertain whether Sirt1 mediates the regulation of Sohlh2 on the interaction between VECs and TNBC cells, Sirt1 inhibitor (EX527) or activator (SRT1720) was utilized in the co‐culture system to confirm that Sirt1‐mediated Sohlh2 regulates the interaction between VECs and TNBC cells. There are five Sohlh2 DNA binding E box sequences (CANNTG) in the promoter region of Sirt1, but the E box sequence through which Sohlh2 exerts its effect needs to be further studied.

Post‐translational modification of proteins is involved in the regulation of protein function by altering protein activity, stability and interaction with other molecules [36, 37]. The study of post‐translational modification of Sohlh2 protein may provide experimental basis for the future treatment of TNBC lung metastasis targeting Sohlh2. Our laboratory previous studies have demonstrated that TRIM21 mediates the ubiquitination degradation of Sohlh2 in macrophages and inhibits M2 macrophage polarization and TNBC lung metastasis [25]. However, the mechanism of post‐translational modification of Sohlh2 protein remains unclear. The Sohlh2‐interacting protein PRMT5 of Sohlh2 was screened by Co‐IP and mass spectrometry. PRMT5‐mediated Sohlh2 arginine methylation promoted the CUL4B‐mediated ubiquitylation and degradation Sohlh2 in VECs, suggesting that PRMT5‐mediated Sohlh2 arginine methylation may be involved in the regulation of the interaction between VECs and TNBC cells.

PRMT5 is a type II protein arginine Grade‐A transferase that catalyzes the symmetric di‐methylation of the substrate protein arginine [38, 39]. PRMT5 regulates gene transcription through a variety of mechanisms, including changes in chromatin structure, gene transcription, RNA extension, and arginine methylation of splicing‐related proteins, thus participating in the regulation of tumor formation and progression [40, 41, 42]. PRMT5‐mediated methylation of target protein arginine induces the expression of inflammation‐related molecules such as e‐selectin, VCAM‐1, IL‐2, CXCL8/IL‐8, and IκBα [43, 44, 45, 46]. PRMT5 plays an important role in endothelial dysfunction. These findings confirm that PRMT5 plays an important role in tumor metastasis and endothelial dysfunction. In this experiment, overexpressed Sohlh2 in VECs were transfected with PRMT5 overexpressed plasmid and co‐cultured with TNBC cells. We found PRMT5 weakened the inhibition of Sohlh2 on the adhesion of TNBC cells to VECs and trans‐endothelial migration, increased TNBC cell‐induced EC inflammation, permeability increase and senescence. These results suggest that PRMT5 may be involved in the regulation of the interaction between TNBC cells and VECs by promoting the arginine methylation and ubiquitination degradation of Sohlh2. In the following experiments, we will use endothelial Sohlh2 CKI mice to injecting PRMT5 inhibitors into the tail vein to detect the role of PRMT5‐mediated Sohlh2 arginine methylation in TNBC lung metastasis in vivo, and identify the arginine site of PRMT5‐mediated Sohlh2 methylation by mass spectroscopy. The ubiquitin‐proteasome system is an important pathway to accurately regulate protein stability [47]. In our study, we confirmed that CUL4B is an interacting protein of Sohlh2, promoting the ubiquitination degradation of Sohlh2 protein, and the arginine methylation modification of Sohlh2 mediated by PRMT5 enhanced the ubiquitination and degradation of Sohlh2 catalyzed by CUL4B, suggesting that CUL4B could mediate the ubiquitination and degradation of Sohlh2 on regulating the interaction between VECs and TNBC cells, and TNBC lung metastasis.

In summary, we propose a potential mechanism by which Sohlh2 regulates the interaction between VECs and TNBC cells. PRMT5‐mediated Sohlh2 arginine demethylation destabilizes Sohlhh2 protein to weaken the inhibitory effects of Sohlh2 on TNBC cell adhesion, trans‐endothelial migration and VEC dysfunction induced by TNBC cells. Our findings clarify the role of PRMT5/Sohlh2/Sirt1 signaling pathway in VECs regulates the lung metastasis of TNBC, suggesting its potential relevance to clinical conditions associated with vascular endothelial in TNBC lung metastasis treatments.

## 4 Experimental Section

### 4.1 Cell Lines and Culture

Human umbilical vein endothelial cells (HUVECs), human lung microvascular endothelial cell line‐5a (HULEC‐5a), MDA‐MB‐231, EO771 and HEK293T cells were purchased from the Cell Banks of Type Culture Collection of Chinese Academy of Sciences (Shanghai, China). HUVECs were maintained in Endothelial Cell Medium (ECM, Science Cell). HULECs were cultured in MCDB131 medium. MDA‐MB‐231 and EO771 cells were cultured in DMEM medium. All cells were cultured in medium supplemented with 10% FBS and 1% penicillin/streptomycin at 37°C in a humidified atmosphere with 5% CO_2_. The cell lines were authenticated by short tandem repeat (STR) profiling and tested free of mycoplasma.

### 4.2 RNA Interference and Plasmid Transfection

HUVECs and HULECs Were Transfected With Sohlh2 Lentiviral GV358 or shRNAs (Genechem, China). Stable‐expressing Cells Were Established by Puromycin (25 µg/mL, Solarbio) Selection. Cells Were Harvested After Puromycin Selection for Transfection Efficiency by qPCR and Immunoblotting. For Knockdown of PRMT5 and CUL4B, siRNAs Against PRMT5 (Genechem, China) or CUL4B (shRNA), Were Transfected Into VECs Using Lipofectamine 3000 According to the Manufacture's Protocol. The Target Sequences Selected Are Shown in Table S2

### 4.3 Mice

All animal experiments were approved by the Ethics Committee of School of Basic Medical Science of Shandong University (ECSBMSSDU2018‐2‐36). The *Sohlh2^fl/fl^* mice were constructed by Cyagen using the CRISPR‐Cas9 gene‐editing system. *Tek‐Cre* mice were purchased from Cyagen. *Sohlh2^fl/fl^* mice were crossed with *Tek‐Cre* mice to generate *Sohlh2^fl/fl^ Tek‐Cre* mice (named as, Sohlh2 CKI).

### 4.4 Lung Metastasis Mouse Model

After tamoxifen‐induced Sohlh2 knock in. EO771 cells (5 × 10^5^) were injected into the tail vein with 6‐week‐old female mice to establish the TNBC lung metastasis model. All mice (5 mice per group) were randomly divided into the control and experimental groups. The phenotype was analyzed by a blind investigator. All mice were imaged and sacrificed for analyzing lung metastasis in vivo.

### 4.5 In Vivo Vessel Permeability Assay

Tumor‐bearing Control mice and Sohlh2 CKI mice were injected intravenously with 100 µL of a mixture containing 2 mg/mL FITC‐dextran (sigma, 40 kDa) in PBS. After 30 min, the mice were euthanized, and cardiac perfusion was performed with 10 mL PBS. Lung tissue was fixed with 4% paraformaldehyde at 4°C for 24 h. Then passed through sucrose solutions of 20% for 24 h, and embedded in OCT. Frozen blocks were cut into 10‐µm cryosections. Images were obtained using a Leica confocal microscope. The fluorescence intensity of FITC‐dextran was quantified by using ImageJ software.

### 4.6 Patient Specimens

The human TNBC tissue microarrays (TMAs) containing 80 cases of TNBC specimens, purchased from Shanghai Superbiotek Pharmaceutical Technology Inc. (Shanghai, China). The histopathological diagnose were determined by expert pathologists, and the study complied with the Ethics Committee of School of Basic Medical Science of Shandong University (ECSBMSSDU2018‐1‐019).

### 4.7 Tumor Cell Adhesion Experiments

HUVECs and HULECs were seeded in 24‐well plates, and treated for addition of serum‐free medium containing 10 ng/mL TNFα for 2 h. MDA‐MB‐231 cells labeled with the Cell‐Tracker Green CMDFA were added and cultured in 37°C incubator for 2 h. Then, unadhered MDA‐MB‐231 cells were removed by PBS washing. Cells were fixed with 4% paraformaldehyde solution for 30 min, drained the fixative, washed twice with PBS. An inverted fluorescence microscope was photographed and Image J counted adherent MDA‐MB‐231 cells.

### 4.8 Trans‐Endothelial Migration

Transwell chamber was placed into a 24‐well plate, and 2 × 10^4^ VECs were planted above the chamber. After the cells were fully fused into a monolayer, the culture medium was replaced with serum‐free cells, and 3 × 10^4^ MDA‐MB‐231 cells labeled by the Cell‐Tracker Green CMDFA were added. Cells were cultured in 37°C incubator for 12–16 h. Cells above the chamber were gently removed with cotton swabs, fixed with 4% paraformaldehyde solution for 30 min. Random selected visual fields were photographed under an inverted fluorescence microscope, and MDA‐MB‐231 cells migrated out by Image J statistics.

### 4.9 Conditioned Medium (CM)

TNBC cells were seeded at 3 × 10^5^ per well in 6‐well plate. After the cells have adhered, gently wash them twice with PBS continue to culture in fresh serum‐free medium for 24 h. The Collect the supernatant and then centrifuge it at 3000 rpm, Take out the cells and cell fragments in 5 min and store them at ‐80°C until use as CM.

### 4.10 Senescence‐Associated β‐Galactosidase Staining

For β‐Galactosidase (SA‐β‐gal) staining assay, VECs were seeded in 6‐well plates, and MDA‐MB‐231 cell conditioned medium(CM) was added. After 24 h, the cell culture medium was removed. SA‐β‐gal staining fixative was added and fixed for 20 min. SA‐β‐gal staining solution was added, then incubation in 37°C overnight. The observation of SA‐β‐gal‐positive cells was performed under an inverted microscope.

### 4.11 Permeability Experiment

Transwell chamber was placed in the 24‐well plate, and 3 × 10^4^ VECs were implanted above the chamber. After VECs were completely fused into monolayer, the chamber medium was replaced with MDA‐MB‐231 cell CM and treated for 24 h. The chamber was treated with 40 kDa FITC‐dextran containing 1 mg/mL (FITC) labeled dextran in the cell for 1 h. The excitation wavelength of 488 nm and the emission wavelength of 510 nm were read by microplate reader. The permeability of vascular endothelium was analyzed by detecting the fluorescence intensity of glucan penetrating into the lower part of the cell.

### 4.12 RT‐qPCR

TRIZOL reagent (Invitrogen) was used to extract RNA according to the manufacturer's instructions and was reverse transcribed using reverse transcriptase (YEASEN). QPCR Reactions were performed using Hieff Unicon Universal TaqMan multiplex qPCR master mix (YEASEN). The PCR primers are shown in Table S3. Data were normalized to GAPDH expression in each sample.

### 4.13 Western Blot Analysis

Cells were lysed in RIPA buffer (Solarbio) containing protease inhibitor cocktails (MCE) and Phosphatase inhibitors. The proteins were separated by SDS‐polyacrylamide gel, transferred onto polyvinylidene fluoride membranes (Bio‐Rad) that were blocked with 5% dry milk for 2 h, then incubated with primary antibodies overnight at 4°C, followed by peroxidase‐conjugated secondary antibodies for 1 h at RT. The immune complex was visualized by an enhanced chemiluminescence kit (Millipore). All antibodies used in this study are provided in Table S4.

### 4.14 Multiplexed Immunofluorescent Staining

Multiplexed immunofluorescence staining of TMAs was performed using a four‐color multiplex fluorescent immunohistochemistry kit (absin) according to the manufacturer's instructions. Slides were incubated with the primary antibody (Sohlh2, CD31 or Sirt1) for 1 h, and subsequently incubated with HRP‐conjugated secondary antibody for 10 min after removed primary antibody and washed in TBST buffer. The above procedures were repeated for other antibodies, and antibodies were removed by microwave treatment (1 min 100% power) before another round of staining was performed, then sealed with DAPI. Details of antibodies are described in Table S3.

### 4.15 Cytokine Elisa

The concentration of IL‐1β, IL‐6, IL‐8 and TNFα in the CM or sera was measured with Elisa Kit (ExCell Bio). Co‐culture CM or mice sera was added to 96‐well plates for 2 h according to the manufacturer's instructions. All experiments were done in triplicates, performed three separate times.

### 4.16 ChIP Assay

For the ChIP assay, 1 × 10^7^ VECs were prepared following the manufacturer's instructions (Cell Signaling Technology). Briefly, the chromatin fragments solutions were incubated with 10 µL anti‐Sohlh2 antibody or the rabbit IgG and Protein G magnetic beads at 4°C overnight with rotation state. The precipitated DNAs were eluted, separated by magnetic separator, purified using spin columns, and finally analyzed by qPCR. The specific primer pairs for human Sirt1 promoter were shown in Table S2.

### 4.17 Luciferase Reporter Assay

For the luciferase reporter assay, Sohlh2‐overexpression or knockdown VECs were seeded into 24‐well plates and transfected with 10 ng pGL3‐firefly luciferase plasmids together with 10 ng pRL‐Renilla luciferase plasmid and 200 ng Sirt1 luciferase reporter using Lipofectamine 3000 (Invitrogen). The activities of the luciferases were measured by the Luciferase Reporter Assay system (Promega) according to the manufacturer's instructions. Firefly luciferase activities were normalized to those of Renilla luciferase activities in each sample.

### 4.18 Co‐Immunoprecipitation (Co‐IP)

HEK293T cells were collected 24 h after transfection and lysed in lysis buffer supplemented with a protease inhibitor cocktail (Sigma), and a phosphatase inhibitor cocktail (Sigma). Supernatants were collected and incubated with anti‐Flag antibody and normal mouse IgG (Sigma) as a negative IP control for 2 h. The mixtures were incubated with protein A/G beads (MCE) overnight at 4°C. Then, beads were washed 3 times with lysis buffer and eluted by boiling with 2× SDS loading buffer.

### 4.19 Ubiquitination Assay

To assess the ubiquitination of Sohlh2 in VECs, the cells were transfected with Flag‐Sohlh2, HA‐Ub, CUL4B, PRMT5 or shPRMT5. Whole‐cell extracts were Immunoprecipitated with anti‐Flag and analyzed by immunoblotting with anti‐HA antibody.

### 4.20 Mass Spectrometry

After staining protein in SDS‐PAGE gels with coomassie blue, gel lanes were sliced into different bands. The specific analysis process was performed as the manufacturer's instructions.

### 4.21 Statistical Analysis

All statistical analysis were performed using GraphPad Prism 7. The assays were repeated at least three times, and data are presented as mean ± standard deviation (SD). Unpaired 2‐tailed *t*‐test, Pearson correlation test, and log‐rank test were employed for statistical analysis. One‐way ANOVA was used to analyze the differences between three or more sample groups. *P*< 0.05 considered significant (*, *p* < 0.05; **, *p* < 0.01; ***, *p* < 0.001). More information of the materials and methods is in the Supplementary Materials.

## Author Contributions

J.H. and R.H.Z. contributed to the experiment design. R.H.Z., Y.L.X., F.Q.W., L.L.L., Q.Z., X.N.H., Y.H.N., and Y.L.D. performed experiments and gathered experimental data. R.H.Z. and J.H. wrote the manuscript. All authors read and approved the final manuscript's submission.

## Conflicts of Interest

The authors declare no conflicts of interest.

## Supporting information




**Supporting File 1**: advs74884‐sup‐0001‐SuppMat.docx.

**Supporting File 2**: advs74884‐sup‐0002‐OriginalWesternblots.docx. [Correction added on 18 April 2026 after first publication: Supporting File 1 is updated.]

## Data Availability

The data that support the findings of this study are available on request from the corresponding author. The data are not publicly available due to privacy or ethical restrictions.
