# Supplementary material for: PRMT5/Sohlh2/Sirt1 Signaling Pathway in Vascular Endothelial Cells Modulates Lung Metastasis of Triple‐Negative Breast Cancer
Source: Adv Sci (Weinh). 2026 Mar 23;13(31):e74884. doi: 10.1002/advs.74884 (PMC13588014; doi:10.1002/advs.74884)
Supplement: Supplementary file 1 [file ADVS-13-e74884-s001.docx]

Original western blot of Figure S1

Original western blot of Figure 2

Original western blot of Figure S2

Original western blot of Figure 4

Original western blot of Figure 5

Original western blot of Figure S4

Original western blot of Figure S5

Original western blot of Figure 8

Original western blot of Figure S6

Original western blot of Figure 9
