# Supplementary material for: PRMT5/Sohlh2/Sirt1 Signaling Pathway in Vascular Endothelial Cells Modulates Lung Metastasis of Triple‐Negative Breast Cancer
Source: Adv Sci (Weinh). 2026 Mar 23;13(31):e74884. doi: 10.1002/advs.74884 (PMC13588014; doi:10.1002/advs.74884)
Supplement: Supplementary file 2 [file ADVS-13-e74884-s002.docx]

**Supplementary materials**

Figure S1. Sohlh2 overexpression or knockdown stable VEC lines were successfully constructed. A, B) QPCR assay showed the efficiency of Sohlh2 knockdown in VECs (n=3). C, D) Western blot assay showed the efficiency of Sohlh2 knockdown in VECs. Relative protein expression levels normalized to those of β-actin are shown below each blot. Data are presented as the mean ± SD. Statistical analysis were performed by GraphPad Prism 7 using the unpaired 2-tailed *t* test to detect differences between the groups. ***p* < 0.01, ****p* < 0.001.

Figure S2. The construction of Tie2-specific Sohlh2 knock-in mice. A) Schematic diagram depicting the generation of endothelial cell-specific Sohlh2 knock-in mice. Female *Sohlh2^fl/fl^Tek^CreERT2-^* mice were used as controls. B)The genotypes of the offspring mice were identified by PCR. C) Immunofluorescence assay showed the expression of Sohlh2 and CD31 in lung tissues of mice (Scale bar: 50 μm; n=5). D) Statistical results of Sohlh2 expression in CD31-positive cells (n=5). E) Western blot analysis was used to detect the protein expression of Sohlh2 in Mouse Pulmonary Microvascular Endothelial Cells (MPMECs). F) QPCR assay was performed to evaluate the mRNA level of Sohlh2 in MPMECs (n=3). The values indicate the mean ± SD of three independent experiments. Statistical analysis were performed by GraphPad Prism 7 using the unpaired 2-tailed *t* test to detect differences between the groups. ****p* < 0.001.

Figure S3. Sohlh2 upregulates the expression of Sirt1 and reduces the expression of p-p65. A) mRNAs regulated by Sohlh2 in VECs. B) QPCR analysis was performed to determine the Sirt1 mRNA level in VECs (n=3). C) Immunofluorescence staining was used to detect the expression of Sirt1 in So hlh2 CKI mice (Scale bar: 50 μm; n=5). D,E) NF-κB signaling pathway was enriched by PCR Array in Sohlh2 RNAi VECs. F) QPCR analysis was performed to determine the expression of VEGFa and VCAM1 mRNA levels in VECs with conditioned medium (CM) from MDA-MB-231 cells (n=3). G) Immunofluorescence staining was carried out to detect the expression of p-p65 in VECs with CM from MDA-MB-231 cells (Scale bar: 50 μm; n=3). Data are presented as the mean ± SD. Con and Sohlh2 were compared using the unpaired 2-tailed t-test. For siRNA experiments, differences among siCon, siSohlh2-1 and siSohlh2-2 were analyzed by one-way ANOVA followed by Dunnett’s post hoc multi-comparison test (each siSohlh2 vs siCon). Experiments were repeated at least three times. ***p* < 0.01, ****p*< 0.001.

Figure S4. SRT1720 attenuates the effects of Sohlh2 shRNA on the interaction of VECs and TNBC cells. A-D) The adhesion and trans-endothelial migration of MDA-MB-231 cells were examined in Sohlh2 knockdown VECs with SRT1720 treatment by tumor cell adhesion assay and trans-endothelial migration assay (Scale bar: 50 μm; n=3). E-G) SA-β-gal staining and Western blot analysis were carried out to detect the senescence of Sohlh2 knockdown VECs with SRT1720 addition (Scale bar: 50 μm; n=3). Relative protein expression levels normalized to those of β-actin are shown below each blot. H) Permeability experiment was carried out in Sohlh2 knockdown VECs with SRT1720 co-cultured with MDA-MB-231 cells(n=3). I) The tight junction proteins markers were investigated in Sohlh2 knockdown VECs with CM from MDA-MB-231 cells after SRT1720 added by Western blot analysis. Relative protein expression levels normalized to those of β-actin are shown below each blot. J,K) QPCR and Elisa analysis were performed to determine the expression of inflammatory cytokines in Sohlh2 knockdown VECs with CM from MDA-MB-231 cells after SRT1720 added(n=3). L) Western blot analysis showed the protein levels of p-p65, VEGFa and VCAM1 in Sohlh2 knockdown VECs with CM from MDA-MB-231 cells after SRT1720 added. Relative protein expression levels normalized to those of β-actin are shown below each blot. Data are presented as the mean ± SD. One-way ANOVA analysis followed by Sidak post hoc multi-comparison test was used. Experiments were repeated at least three times. ***p* < 0.01, ****p*< 0.001.

 Figure S5. Knock down Sirt1 attenuates the effects of Sohlh2 on the interaction of VECs and TNBC cells.

A) QPCR assay was performed to detect the mRNA expression of Sirt1 in VECs overexpressed Sohlh2 with transfected Sirt1 knockdown plasmid(n=3). B) Western blot assay was performed to detect the protein expression of Sirt1 in VECs overexpressed Sohlh2 with transfected Sirt1 knockdown plasmid. Relative protein expression levels normalized to those of β-actin are shown below each blot. C) The ability of tumor cells to adhere to VECs was examined by tumor cell adhesion assay (Scale bar: 50 μm; n=3). D) Trans-endothelial migration assay was performed to detect the trans-endothelial migration of tumor cells (Scale bar: 50 μm; n=3). Data are presented as the mean ± SD. One-way ANOVA analysis followed by Sidak post hoc multi-comparison test was used. Experiments were repeated at least three times. ****p* < 0.001.

Figure S6. PRMT5 overexpression VEC lines were successfully constructed. A, B) QPCR assay showed the efficiency of PRMT5 overexpressed in VECs(n=3). C, D) Western blot assay showed the efficiency of PRMT5 overexpressed in VECs. Relative protein expression levels normalized to those of β-actin are shown below each blot. The values indicate the mean ± SD of three independent experiments. Statistical analysis were performed by GraphPad Prism 7 using the unpaired 2-tailed *t* test to detect differences between the groups. ****p* < 0.001.

**Table S1: The correlation of Sohlh2 expression in TNBC VECs and clinical characteristics**

**Table S2:**

The target sequences selected are shown as follows:

| siRNA | target sequences (5’-3’) |
| --- | --- |
| siSohlh2-1 | GCUCCAAUUCCUGACUAAUAC |
| siSohlh2-2 | UCUCCUGCCGUUAUGGCCCAGAUUA |
| shPRMT5-1 | CCCATCCTCTTCCCTATTAAG |
| shPRMT5-2 | GCCCAGTTTGAGATGCCTTAT |
| shCUL4B | gataagcctaaattaccagaa |

**Table S3:** Primer sequence for qPCR

| Gene | Forward Primer | Reverse Primer |
| --- | --- | --- |
| *GAPDH* | GAAAGCCTGCCGGTGACTAA | AGGAAAAGCATCACCCGGAG |
| *TNFα* | AAAACAACCCTCAGACGCCA | TCCTTTCCAGGGGAGAGAGG |
| *Sirt1* | TCCTACTGGCCTGAGGTTGA | AAAGAGCGAATCCACTGGCA |
| *IL-1β* | TCGCCAGTGAAATGATGGCT | GGTCGGAGATTCGTAGCTGG |
| *IL-6* | CCACCGGGAACGAAAGAGAA | GAGAAGGCAACTGGACCGAA |
| *IL-8* | CACTGCGCCAACACAGAAATTA | TTCTCAGCCCTCTTCAAAAACT |
| *VEGFa* | GCTGTCTTGGGTGCATT | GCAGCCTGGGACCACTTG |
| *VCAM1*  *Sirt1*-ChIP | TGGATAATGTTTGCAGCTTCTCA  ATTTGGCTGCACTACACGCT | CGTCACCTTCCCATTCAGTG  CGGGCCAGACCACAACACTA |

**Table S4:** The list of antibodies used for Western blot analysis

| Parameter | Producer | Cat# |
| --- | --- | --- |
| Sohlh2 | Novus | NBP2-20453 |
| CD31 | Bosterbio | A01513-3 |
| Sirt1 | Affinity | DF6033 |
| β-actin | Affinity | T0022 |
| ZO-1 | Abcam | Ab96587 |
| VE-cadherin | Affinity | AF6265 |
| Occludin | Affinity | DF7504 |
| p16  p21 | Affinity  Affinity | BF0580  AF6290 |
| ET-1  p65  p-p65  VEGFa  VCAM1  PRMT5  CUL4B  HA  His  Flag  sDMA | Affinity  CST  CST  Affinity  Affinity  CST  Proteintech  CST  sigma  sigma  PTM BIO | DF6125  #8242  #3033  AF5131  DF6082  #79998  12916-1-AP  #3724  #2365  F1804  ABT-117106 |
